# Deeply Implanted Conformal Antenna for Real-Time Bio-Telemetry Applications

**DOI:** 10.3390/s24041170

**Published:** 2024-02-10

**Authors:** Ladislau Matekovits, Farzad Mir, Gianluca Dassano, Ildiko Peter

**Affiliations:** 1 Department of Electronics and Telecommunications, Politecnico di Torino, 10129 Turin, Italy; ladislau.matekovits@polito.it (L.M.); gianluca.dassano@polito.it (G.D.); 2 Istituto di Elettronica e di Ingegneria dell’Informazione e delle Telecomunicazioni, National Research Council of Italy, 10129 Turin, Italy; 3 Faculty of Electronics and Telecommunications, Politehnica University Timișoara, 300006 Timișoara, Romania; 4 Department of Electrical and Computer Engineering, University of Houston, Huston, TX 77204, USA; fmir@cougarnet.uh.edu; 5 Department of Industrial Engineering and Management, Faculty of Engineering and Information Technology, George Emil Palade University of Medicine, Pharmacy, Science, and Technology of Tirgu Mureș, 540139 Tirgu Mureș, Romania

**Keywords:** bio-electromagnetic, bio-telemetry, conformal antennas, implanted antenna, in-body microstrip antenna

## Abstract

The design and experimental verification of a deeply implanted conformal printed antenna is presented. The hip implant acts as the ground plane for a coaxial-cable-fed trapezoidal radiator designed to transmit biological signals collected within the body by proper biosensors. The arrangement, consisting of a metallic (or equivalent) hip implant, bio-compatible gypsum-based dielectric, and conformal radiator, was tested when the hosting 3D-printed plastic bone was immersed in tissue-like liquid contained in a plastic bucket. The dimensions of the set-up are similar to a human leg. Matching and radiation characteristics are presented in the industrial, scientific, and medical (ISM) frequency band (2.4–2.5 GHz), showing the feasibility of the proposed arrangement.

## 1. Introduction

Bio-telemetry is becoming ever more diffused in the modern medical world. It allows for the remote monitoring of patients, improving their personal well-being, as monitoring of their physiological signals can be carried out in the home environment and reduce hospitalization time, hence cutting costs for the healthcare system. Measurements of patients’ physiological signals can be performed by means of either wired or wireless devices [1,2,3]. The results of the measurements of the signals in the form of current and/or voltage are transmitted through the proper system and received by the appropriate control devices [4,5] for re-transmission or elaboration.

The idea of implanting a sensing device with antennas and wireless functionality into bio-tissues (e.g., vessels or bones) has been considered for over a decade. Different forms of antennas folded into limited cylindrical/conical space have been thoroughly investigated [6]. 

Recently, crucial developments have been achieved in this field; the latest of these are based on the higher precision obtained due to the use of implantable medical devices (IMDs). These sensor-equipped apparatuses can collect data closer to the area of interest; therefore, a more precise description of the relevant physiological signal(s) can be taken. A signal has to be sent outside the body; one of the first and most common designs is based on low-frequency inductive links [7]. This method has been used for a long time, but due to some significant limits such as low data rates, restricted range of communication, and increased sensitivity to inter-coil positioning [8], a new approach, known as radio-frequency-linked IMDs, has been suggested as a solution to overcome these issues [9]. 

IMDs are quite common, and their use has contributed to a remarkable increase in the quality of life, and consequently, an increase in human well-being. Nowadays, these devices are widely used in various medical fields: monitoring body temperature, cardioverter defibrillators [10], checking blood glucose [11], monitoring the pH level of skin samples [12], cochlear [13] and retinal devices [14], wireless cardiac pacemakers [15], and teeth-implantable devices [16]. Many other innovations are under different stages of development. 

Frequency bands dedicated to bio-telemetry IMDs include 402−405 MHz (ITU-R Recommendation SA.1346.) [17,18], 868−868.6 MHz, 902.8−928 MHz, and 2.4−2.5 GHz. For the investigations discussed in this paper, the last frequency range mentioned above has been considered. It is known as an ISM frequency band [19]. 

The aim of the study was to describe a low-profile implanted antenna with a hip implant as the ground plane. The proposed configuration’s main advantages are multifold. The antenna is located on the implant; thus, no extra space with respect to the implant itself is needed. In addition, it is meant to be inserted into the human body concurrently with the implant, so no extra surgery is necessary. The antenna is thought to transmit collected bio-signals nearby to the implant, a necessary and important aspect during rehabilitation after the surgery. Biosensors such as pH, temperature, humidity, and Ca-ion concentration can be located near the wounded tissue and linked to an electronic device. This latter mechanism (i) controls the sensors that collect data, (ii) refines these inputs, and (iii) sends them to the antenna. The presence of sensors close to the tissues of interest leads to the real-time detection of aspects such as implant rejection, infections, and gum recession. The circuitry can be located inside the metallic implant, which also acts as a shield. 

Bio-compatibility, miniaturization, and patient safety are the most challenging aspects when designing IMDs. Bio-compatibility refers to both dielectrics and conductors. Though the considered metal is almost transparent to the numerical design, the limitations of available dielectrics are more critical. The relative dielectric constant and losses must be considered to tune the resonant frequency of the antenna within the body. The considered technology determines the porosity, possible thickness, and other medical characteristics, e.g., capability to connect to bone (ossification) [20]. 

The importance of bio-compatible materials used for implantable antennas has absorbed scientists’ attention. The main purpose is to make sure that the implantable antennas are perfectly covered by these materials to restrain the biological tissue exposed to the dielectric material of the antennas. The main problem arises because of the conductivity of biological tissue, resulting in causing short circuits with the implantable antenna. This is hazardous and may damage the human tissue, generate infections, and render the antennas useless.

There are two main methods to avoid the aforementioned problems. The first is the use of implantable devices with bio-compatible substrates. Ti-based alloys are largely employed for medical implants in orthopedic applications, thanks to their brilliant bio-compatibility and osseointegration capacity, combined with good mechanical properties. Recent research has focused on the development of Ti-based alloys containing a high level of beta phase, in order to lower the Young’s modulus of the alloy and to arrive at a Young’s modulus that is close to that of human cortical bone (E = 15–30 GPa), avoiding a stress-shielding effect. In this context, some of the authors of the present paper carried out investigations in order to develop a beta phase containing quaternary Ti-based alloys, such as Ti-10Nb-10Zr-5Ta, Ti-20Nb-20Zr-4Ta, and Ti-29.3Nb-13.6Zr-1.9Fe. Another way to preserve bio-compatibility is the use of bio-compatible encapsulation, when the implantable device is covered with a thin layer of bio-compatible material [21], as reported in [22]. Here, enhancement of the bio-compatibility of the metallic alloy was achieved with an electrochemical anodization method. 

Some well-known materials are categorized into two groups, as follows. The first group consists of ceramic materials such as Teflon (*ϵ_r_* = 2.1, tan *δ* = 0.001), MACOR (*ϵ_r_* = 6.1, tan *δ* = 0.005), alumina (*ϵ_r_* = 9.4, tan *δ* = 0.006) [18], and zirconia with *ϵ_r_* = 10, tan *δ*= 0.0005 [23].

The second group consists of polymeric materials. The most well-known are Parylene-C, characterized by *ϵ_r_* = 2.95, tan *δ* = 0.013 [24], Polymaid with *ϵ_r_* = 4.3, tan *δ*= 0.004, and Polypropylene (*ϵ_r_* = 2.55, tan *δ* = 0.003) [25]. Hydroxyapatite [26] is another bio-compatible substance. Gypsum is a familiar bio-material which is non-metallic and consists of hydrated calcium sulfate (*CaSO*_4_·2*H*_2_*O*). This material has received attention for several decades in a variety of uses, especially in dentistry. Moreover, because of the osteoconductive properties of *CaSO*_4_, gypsum is widely used in bone creation. In addition, the bio-resorption (safe for the human body) of gypsum is another advantage for this bio-material that makes it attractive [27]. The values above refer to the ISM frequency band.

In the present design, miniaturization was not essential. Some of the implants, e.g., hip or knee, are large by default. This usually metallic part represents an ideal ground plane for antennas. Combining them with low-space-occupation configurations, e.g., printed microstrip antenna, the radiator can be inserted in the body with zero volume requirement, which is the major advantage of the proposed solution. In some cases, as for the knee implant, these ground planes are close to the skin, but, in other cases, as for a hip implant considered in this study, they are deeply located within the body. This antenna location raises other challenging issues, as the high water content of the body induces high losses.

After designing and fabricating the implantable antennas, the next step is to test the final model. The measurements can be done by two main methods: in vitro [28], when the model is tested in the laboratory in a similar environment to the living body environment but with no use of any living tissue, and in vivo, when the model is tested on living organisms [29]. In this study, the measurement was carried out in the laboratory by means of a quasi-in vitro technique defined in one of the following sections, so no living tissue was involved.

This research topic has already been considered by some of the authors in previous publications; here, the main differences with respect to the disseminated configurations are summarized, aiming to highlight the advancement in different terms such as feasibility or performance. In [30], a cylindrical implant was considered, and the mutual coupling reduction between two printed radiators was investigated. The antenna was embedded in a Polydimethylsiloxane (PDMS) layer located between the bone and muscle. The radiation mechanism of a recess-fed conformal patch antenna was investigated in [31]. The novelty of the present study is in considering a slightly modified conical shape implant, similar to a commercial one, supporting a trapezoidal printed antenna. The configuration was numerically simulated and prototyped. This latter has been measured in terms of matching and radiation pattern. This study represents a substantial advancement with respect to the state of the art [32,33,34,35,36].

This paper is organized as follows. The antenna design is discussed and the associated numerical results are presented in Section 2. Section 3 is devoted to the presentation of the prototyping steps, description of the measurement set-up, presentation of the experimental results and their comparison with the numerical data. Conclusions are presented in Section 4.

## 2. Methods

The theoretical implant is of a conical shape fully immersed in a cylindrical bone of radius *R_bone_*. The bone is surrounded by a muscle layer and a fat layer that is externally bounded by the skin. The radii of the different interfaces are denoted as follows: *R_mf_* (muscle–fat), *R_fs_* (fat–skin), and *R_s_* (skin–air). To perform experimental validation of the proposed configuration avoiding the use of any in vivo conditions, the configuration was slightly modified, as described below.

### 2.1. Geometries of the Implant, Dielectric Layer, and Bone

**Figure 1:**
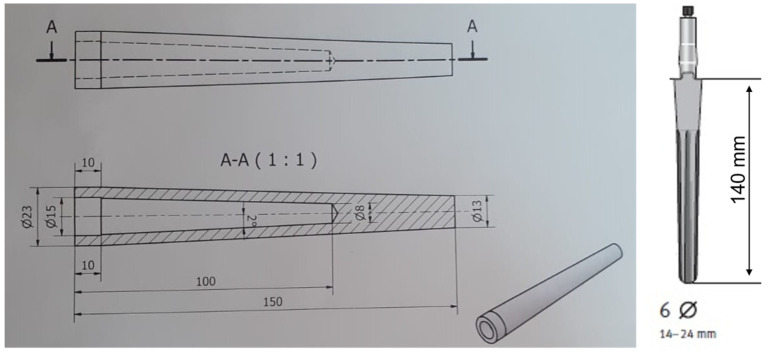
Technical drawing of the geometry of the implant (**left**); geometry of a commercial implant [38] (**right**).

The considered geometry is shown in Figure 1 (left). It is akin to a commercial implant, reported in Figure 1 (right).

It was found that the spurious radiation from the feeding microstrip influences the radiation pattern (RP). The cylindrical implant was substituted by a conical one in [36], where a turn-style antenna was presented achieving circular polarization. The two orthogonal dipoles were located at the bone–muscle interface. In the present solution, the antenna positioning is more realistic, as the antenna is located on the surface of the implant; therefore, no additional intervention is required during the surgery. In the present investigation, a single conformal patch was considered, having a conical shape implant as the ground plane. Feeding is through a coaxial cable, going through the implant and the dielectric layer that covers it. Even if, from the electromagnetic point of view, it is not significant, the metallic implant and antenna can be realized, for example, by a bio-compatible Ti-alloy, e.g., [37].

The implant in Figure 1 (left) is 1 mm *slimmer* along the overall length with respect to the commercial one, with this thickness used for a bio-compatible dielectric supporting the printed antenna. The thickness of 1 mm (in diameter) was equally subdivided between the metal implant and bone, i.e., a slimmer implant, and a larger hole in the bone for implantation was considered. Additionally, an empty internal part of the implant was thought to be capable of hosting future electronics (not considered here), and a coaxial cable fed the antenna. In addition, the corrugated surface of the commercial implant was substituted with a smooth one.

### 2.2. The Simulation Environment

Commercial electromagnetic field simulation software, more precisely the Computer Simulation Technology Suite [39], was used for the simulations, targeting resonance at the central frequency of the ISM band. Two small changes were considered in relation to the literature. (i) A different dielectric constant of the bone was considered, as it was prototyped by a 3D printer (see below), and (ii) with respect to the in vitro case, it consisted of the substitution of the fat and skin layers by a single plastic layer, corresponding to the container of the tissue-like liquid. This set-up is named quasi-in vitro.

**Figure 2:**
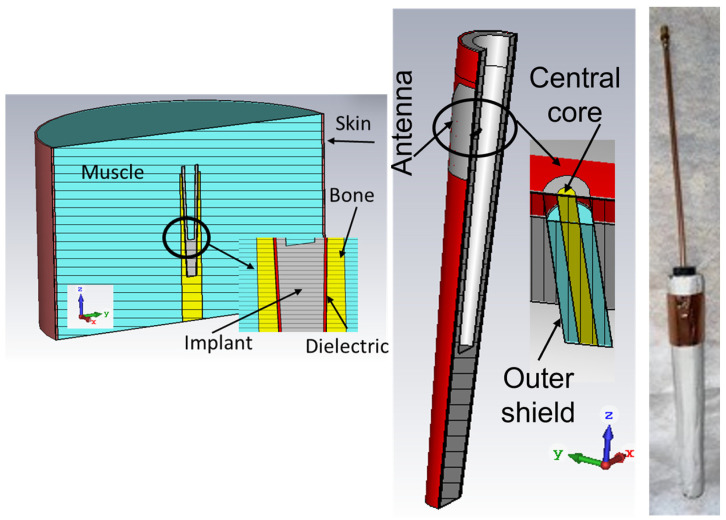
CAD model of the implant immersed in the body implemented in CST (**left**), antenna detail (**center**), and implant with dielectric layer and antenna artwork (**right**).

The above changes would not affect the target to prove the feasibility of the implanted system; only the dimensions of the patch would be slightly altered to still resonate at 2.45 GHz, due to the different dielectric constants of the different layers. Thus, the geometry in Figure 2 was implemented. The dielectric constant of the different layers and leading dimensions are reported in Table 1. The limits for the muscle correspond to the measured values (see below).

As can be observed, the fat layer among the muscle and skin is missing, as the simulations were performed according to the quasi-in vitro conditions stated above, which related to the laboratory set-up used for the measurements (see below). To anticipate this multilayer geometry, the bone was prototyped by a 3D printer (Plastic-2), the muscle tissue-like liquid was synthesized according to [19,40] and measured/characterized experimentally, while the skin–fat layers were substituted with a plastic bucket (of a slightly conical shape, similar to a leg) of Plastic-1 material. The dielectric layer of thickness of *t_d_* = 1 mm all along the implant length consisting of gypsum [27,41] was chosen because of the similar structure of bone (high calcium content of both materials) and low cost. 

**Figure 3:**
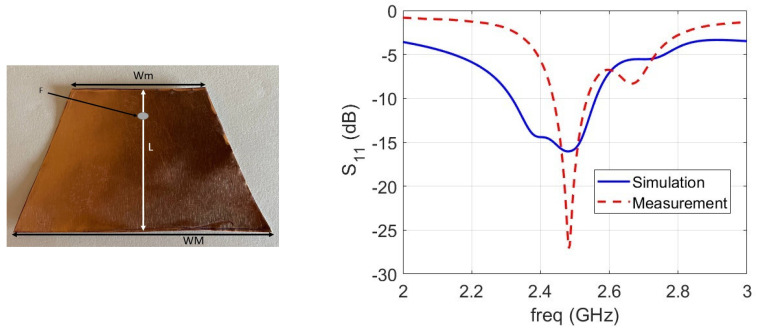
Leading dimensions of the antenna geometry (**left**). Comparison between the matching S11 of the antenna: simulation (continuous line) vs. measurement (dashed line) (**right**).

The antenna consists of a trapezoidal metallization (see Figure 3, left). Its dimensions were determined by optimization using a CST internal genetic algorithm routine; 4 variables were considered, namely patch length (L), shorter (Wm) and longer (WM) patch sizes, and location (F) of the feeding point with respect to the patch edge toward the top of the implant (larger part). The trapezoidal shape adds a further degree of freedom that can compensate, for example, for the curvatures of the metallic surface [42,43]. The cost function components were resonant frequency, impedance bandwidth, and efficiency bandwidth. For assembly reasons, the location of the feeding point was fixed, and the patch was moved along the axial direction, corresponding to changing the value of parameter F. A hole of 1 mm diameter with the center at 43 mm from the top of the implant was considered to allow the passing of the central conductor of a standard fully modeled SMA connector through the implant, used to feed the patch. The CAD model of the dielectric-covered implant hosting the antenna is shown in Figure 2 (right). The matching of the geometry for L = 33.5 mm, Wm = 22 mm, WM = 56 mm, and F = 5.5 mm is reported in Figure 3 (right).

### 2.3. Prototyping

The prototyping process was carried out in different steps.

#### 2.3.1. D Printing

**Figure 4:**
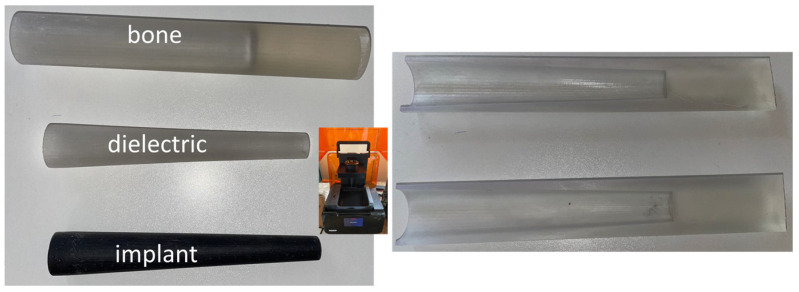
Photograph of the implant, dielectric layer, and bone geometry printed by 3D technology. Inset: 3D printer device (**left**) and the 3D-printed die. The two parts are identical (**right**).

The implant, dielectric, and bone were realized using 3D printer technology. A photograph of them is shown in Figure 4. A special kind of plastic material (available in two different forms, black and clear) was used to print them. These structures were printed with a “3 Form” 3D printer (in the inset). In a second step, the plastic implant was covered by a copper sheet. From an electromagnetic point of view, this corresponds to a metallic implant. Even if a metallic geometry was also realized, the lighter, copper-covered plastic one was used during the measurements, facilitating the rotation during the measurements (see below). 

The bone is a cylindrical structure with a conical hole inside to host the coated implant equipped with the antenna. Its 3D-printed version would be used for the experimental validation, to avoid the use of any living tissue. The die was also 3D-printed. Its dimensions are equal to that of the bone geometry; it consists of two identical parts aimed to be used as a mold to realize a uniform dielectric layer around the implant to fully fit the “hole” in the bone (see Figure 4 (right)).

#### 2.3.2. Preparation of the Antenna Sample

Half of the mold was filled with gypsum, along with the metallic implant. This was covered by the second half of the mold, allowing excess gypsum to be expelled. Before doing so, a *ϕ* = 1 mm diameter hole was drilled at the proper location of the feeding. It would serve as the passage for the semi-rigid coaxial cable used to feed the patch antenna. Once the gypsum had solidified, the mold was eliminated, and the gypsum was cleaned from the hole. In the present investigation, the trapezoidal patch was realized separately and positioned on the conical surface of the gypsum with plastic tape. In the actual realization, a deposition can be used. The feeding semi-rigid coaxial cable was inserted in the cavity inside the implant, and, by hand, its central conductor soldered with tin to the patch. A photograph of the specimen is shown in Figure 2 (right).

### 2.4. Experimental Set-Ups

The experimental part of the investigation had target measurements of the (i) dielectric constant of the self-prepared tissue-like liquid, (ii) matching, and (iii) RP. The experimental set-up used for these quantifications and the procedure used during the evaluations are described below.

## 3. Results and Discussion

### 3.1. Measurement of the Dielectric Characteristics of the Tissue-like Liquid

The tissue-like liquid associated with the muscle was prepared according to references in Section 2. The following mixture was prepared: around 4 l deionized water, 350 g sugar, and 150 g salt, which were combined. In this composition, sugar and salt play a key role in the permittivity (*ϵ_r_*) of the liquid. By increasing the amount of sugar, *ϵ_r_* rises, but it reduces when the salt concentration increases. However, it should be noted that the amount of sugar and salt affects the losses. The fine-tuning of the composition was completed following the measurements of the dielectric characteristics of the liquid using a Keysight Technology P9375A set-up.

**Figure 5:**
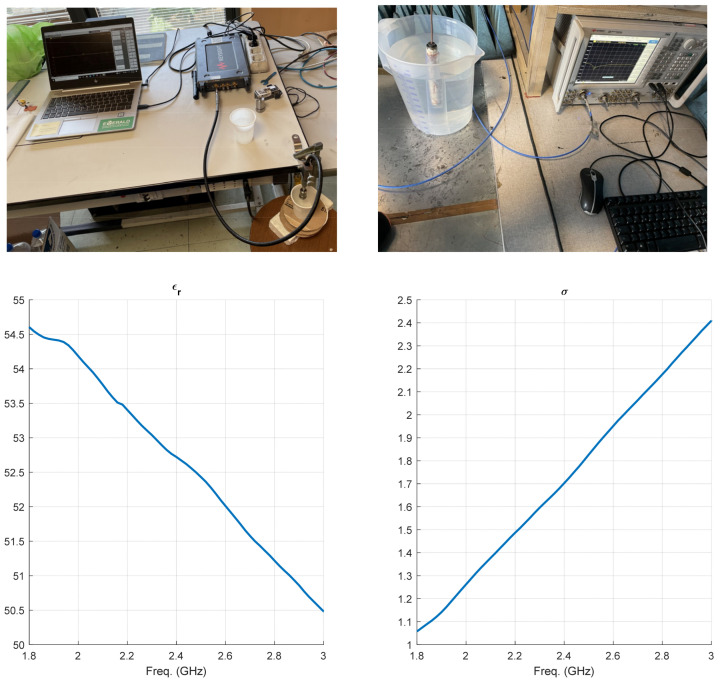
System to measure the dielectric constant of the tissue-like liquid (**top left**), and the quasi-in vitro configuration (**top right**). Measured relative dielectric constant (**bottom left**) and conductivity S/m (**bottom right**).

The measurements were based on the experimental evaluation of the capacity between the two ends of the meter, when the space between them was filled by the liquid. Numerical elaboration considering dispersive effects was performed by in-house software developed in the Matlab environment and tested on commercial samples. A photo of the system is shown in Figure 5 (top), connected to a network analyzer that in turn connects to a PC hosting the code. The values of the complex dielectric constant, in Figure 5 (bottom), were saved in an ASCII file, later used in the simulations carried out in CST. They refer to 400 mg of commercial sugar melted in 1000 mL of demineralized water. In the interested 2.4–2.5 GHz frequency band, the values of ε_r_ and σ varied between 52.7 and 54.5 and between 1.7 and 1.85, respectively.

### 3.2. Measurement of the Antenna Matching

A photo of the antenna in the quasi-in vitro configuration is shown in Figure 5 (top). It shows the building used for optimizing the antenna sizes for this actual set-up. Measured matching is compared with the simulated data in Figure 3. A good agreement can be observed, as the two resonances are present for both sets of data in the interested frequency band. The difference in magnitude was related to the losses in the tissue-like liquid that was used. The losses in the synthesized tissue-like liquid depend on the amount of sugar and salt. In addition, the water content of living tissue depends on age, gender, medical history, etc.

### 3.3. Measurement of the Antenna Gain and RP

**Figure 6:**
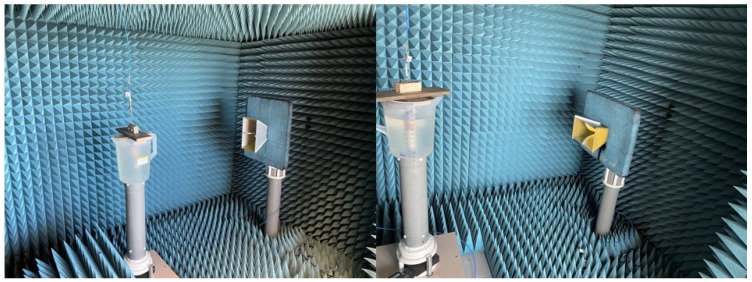
RP measurement set-up for the embedded antenna: cross-polarization (**left**) and co-polarization (**right**) components.

Antenna RP and gain measurements were carried out in an anechoic chamber using the substitution method. Two identical R&S HF906 double-ridged horn antennas were considered for the reference measurement carried out by a Keysight N5227A Vector Network Analyzer (VNA). The distance between the two horn antenna apertures was set to 1.4 m, and 201 frequency points in the 2–3 GHz band were considered. Then, one of the horns was substituted by the antenna immersed in the tissue-like liquid. The bucket was placed on a turning table, having the antenna center at the same height as the center of the aperture of the antenna for the reference measurement. The turning table can be rotated in the [−180, 180] azimuthal angular interval around the symmetry axis of the implant with a step of 1° by a step-by-step motor, controlled by software developed in Labview. The measured values of the transmission coefficient (S21) were recorded. The post-processing performed in Matlab considered the gain of the horn antenna, spatial attenuation, and power transmission coefficient, accounting for the earlier measured matching. A photo of the system is shown in Figure 6 (for both co- and cross-polarization).

**Figure 7:**
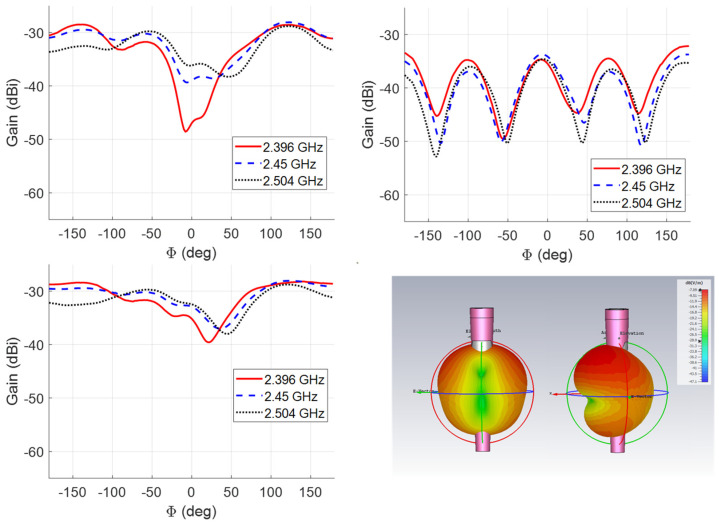
H-field RP: co-polarization (**top left**), cross-polarization (**top right**), total field (**bottom left**), and simulated 3D pattern (**bottom right**).

The measured RPs at the limits of the interested band and at the central frequency of 2.45 GHz in the *H* (*x*0*y* with respect to the coordinate system indicated in Figure 2) plane of the antenna are reported in Figure 7. The difference in amplitude for the two components, co- and cross-polarization, is around 10 dB. This is due to the trapezoidal shape that generates higher azimuthal currents than a square/rectangular patch, as currents are also flowing in the azimuthal direction. Moreover, the propagating electromagnetic field from the source outward encounters different layers and, according to the description of the mechanism in [31], the TE and TM propagation are affected differently. In the present investigation the polarization of the radiated field has not been inserted in the optimization process, but in case this aspect can be considered as a further degree of freedom, also to get circular polarization.

A quite stable pattern in the considered frequency band can be observed, with a reduction in the gain at the lower limit. The higher value of the field in the non-broadside direction is due to the generated surface waves in the gypsum dielectric, according to the discussion in [31]. However, this phenomenon guarantees a relatively stable wide angular coverage; therefore, the relative location of the patient and external receiver does not affect the quality of the overall system. The null in the radiation pattern for phi = 180° is due to the cancellation of the radiation of the two oppositely propagating surface waves going around the structure.

## 4. Conclusions

The design and experimental validation of a deeply implantable smart orthopedic device aiming to send biological signals from inside a human body in the ISM frequency band are discussed. The real-life conical, large implant was covered with bio-compatible dielectric, gypsum, supporting a low-profile conformal trapezoidal radiator fed by a semi-rigid coaxial cable. For validating the simulation results, a prototype was subjected to experimental validation. The explored structure was bounded with a bone made of plastic that was immersed in a mixture of water, sugar, and salt, mimicking human muscle tissue. The aspect of the overall arrangement is like an adult leg. The experimental data match the numerical data in terms of wideband and radiation pattern.

## Figures and Tables

**Table 1:** Dimensions of different parts of the structure.

Tissue	Material	Radial Extension	Length	Rel. Diel.
		(mm)	(mm)	Constant
Skin–Fat	Plastic-1	200–203	257	2.2
Muscle	Liquid	15–200	257	52.7–54.5, σ = 1.7–1.85
Bone	Plastic-2	15	200	3.4
Dielectric	Gypsum	top: 11.5–12.5 bottom: 6.5 4–7.5	140	2.7
Implant	PEC	top: 11.5/bottom: 6.5	140	-

## Data Availability

The data are contained within the article. The data that support the findings of this study are available from the corresponding author upon reasonable request.
